# Assessment of Nocturnal Autonomic Cardiac Imbalance in Positional Obstructive Sleep Apnea. A Multiscale Nonlinear Approach

**DOI:** 10.3390/e22121404

**Published:** 2020-12-12

**Authors:** Daniel Álvarez, C. Ainhoa Arroyo, Julio F. de Frutos, Andrea Crespo, Ana Cerezo-Hernández, Gonzalo C. Gutiérrez-Tobal, Fernando Vaquerizo-Villar, Verónica Barroso-García, Fernando Moreno, Tomás Ruiz, Roberto Hornero, Félix del Campo

**Affiliations:** 1 Pneumology Department, Río Hortega University Hospital, 47012 Valladolid, Spain; caroyodo@saludcastillayleon.es (C.A.A.); jfrutosar@saludcastillayleon.es (J.F.d.F.); acrespos@saludcastillayleon.es (A.C.); acerezoh@saludcastillayleon.es (A.C.-H.); fmorenot@saludcastillayleon.es (F.M.); truizal@saludcastillayleon.es (T.R.); 2 Biomedical Engineering Group, University of Valladolid, 47011 Valladolid, Spain; gguttob@gmail.com (G.C.G.-T.); fervaquerizo@gmail.com (F.V.-V.); veronica.barroso@gib.tel.uva.es (V.B.-G.); robhor@tel.uva.es (R.H.); 3 Centro de Investigación Biomédica en Red en Bioingeniería, Biomateriales y Nanomedicina (CIBER-BBN), 47011 Valladolid, Spain

**Keywords:** positional apnea, cardiac dynamics, heart rate variability, nonlinear analysis, multiscale entropy

## Abstract

Positional obstructive sleep apnea (POSA) is a major phenotype of sleep apnea. Supine-predominant positional patients are frequently characterized by milder symptoms and less comorbidity due to a lower age, body mass index, and overall apnea-hypopnea index. However, the bradycardia-tachycardia pattern during apneic events is known to be more severe in the supine position, which could affect the cardiac regulation of positional patients. This study aims at characterizing nocturnal heart rate modulation in the presence of POSA in order to assess potential differences between positional and non-positional patients. Patients showing clinical symptoms of suffering from a sleep-related breathing disorder performed unsupervised portable polysomnography (PSG) and simultaneous nocturnal pulse oximetry (NPO) at home. Positional patients were identified according to the Amsterdam POSA classification (APOC) criteria. Pulse rate variability (PRV) recordings from the NPO readings were used to assess overnight cardiac modulation. Conventional cardiac indexes in the time and frequency domains were computed. Additionally, multiscale entropy (MSE) was used to investigate the nonlinear dynamics of the PRV recordings in POSA and non-POSA patients. A total of 129 patients (median age 56.0, interquartile range (IQR) 44.8–63.0 years, median body mass index (BMI) 27.7, IQR 26.0–31.3 kg/m^2^) were classified as POSA (37 APOC I, 77 APOC II, and 15 APOC III), while 104 subjects (median age 57.5, IQR 49.0–67.0 years, median BMI 29.8, IQR 26.6–34.7 kg/m^2^) comprised the non-POSA group. Overnight PRV recordings from positional patients showed significantly higher disorderliness than non-positional subjects in the smallest biological scales of the MSE profile (*τ* = 1: 0.25, IQR 0.20–0.31 vs. 0.22, IQR 0.18–0.27, *p* < 0.01) (*τ* = 2: 0.41, IQR 0.34–0.48 vs. 0.37, IQR 0.29–0.42, *p* < 0.01). According to our findings, nocturnal heart rate regulation is severely affected in POSA patients, suggesting increased cardiac imbalance due to predominant positional apneas.

## 1. Introduction

Changes in sleeping position are known to affect the occurrence of apneic events and, consequently, the severity of obstructive sleep apnea (OSA) [1]. In this regard, a number of studies described a group of OSA patients showing a higher rate of obstructive respiratory events in the supine sleeping position [1,2,3,4,5]. These patients suffer from positional OSA (POSA), which is a major sleep apnea phenotype [5,6].

The prevalence of POSA varies among studies, mainly due to the use of different rules for scoring sleep events and the use of different characteristics for identifying positional patients. Recent studies reported a high prevalence of POSA among sleep apnea patients, with estimates ranging from 56% to 75% [3,4,7]. Regarding the criteria used to diagnose POSA, there is not a consensus. Cartwright first defined POSA patients as those showing just an apnea-hypopnea index (AHI) in the supine position at least twice the AHI in the non-supine position [1]. However, several amendments have been proposed since then [4,5,8]. Recently, Frank et al. have proposed a novel classification system, the Amsterdam POSA classification (APOC), aimed at identifying an increased number of patients more likely to benefit from positional therapy [9].

Due to gravitational effects, collapsibility of the upper airway increases in the supine position, leading to more frequent, prolonged and severe apneic events [1,10,11]. Despite the apparently higher severity of respiratory disturbances in the supine sleeping position, few studies investigated the differences between positional and non-positional patients, and findings were often contradictory. A lower total AHI, fewer symptoms and less comorbidity are commonly reported in positional patients [12,13]. On the contrary, Oksenberg et al. [14] reported that, in the supine position, oxygen desaturations were deeper, and the tachycardia and bradycardia cyclic pattern at the end of apneic events were more severe than in non-supine sleeping positions. Therefore, further research is needed to gain insight into the effects of positional apneas. Particularly, as OSA patients show increased risk of suffering from cardiovascular diseases [15,16,17], better understanding of the characteristics of POSA patients concerning heart rate modulation would be beneficial in the management of the disease [18]. In this regard, a recent study by Byun et al. [19] analyzed the differences in cardiac autonomic activity between non-POSA and POSA patients. They reported higher parasympathetic activity linked to position-dependent sleep apnea. Nevertheless, cardiac dynamics were analyzed in a short period (5 min) while patients were awake, thus not enabling them to assess the actual influence of positional apneas on cardiac modulation during the whole night. Similarly, Flevari et al. investigated the heart rate variability (HRV) dynamics of positional patients in the N2 sleep stage in the supine and non-supine positions, reporting augmented cardiac indexes while sleeping in the supine position [20]. However, no differences in overall cardiac modulation between POSA and non-POSA patients were assessed.

Heart rate variability (HRV) has been widely used to assess cardiac dynamics during sleep and in the presence of sleep disorders. Particularly, the analysis of HRV has been found to provide relevant information on the autonomic imbalance linked to OSA [21]. In the same regard, recent studies suggest that nocturnal HRV analysis is a useful tool to assess the efficacy of the most common therapies in the context of OSA treatment [22,23]. The signal power in the conventional frequency bands of the HRV spectrum has been predominantly used due to its close relation with sympathetic and parasympathetic modulation of the heart rate [21,24,25]. Nevertheless, nonlinear analysis has been found to outperform common frequency domain indices in the context of OSA [26,27,28,29]. Particularly, entropy measures such as sample entropy (SampEn) and multiscale entropy (MSE), which quantify disorderliness or irregularity and complexity of a time series, recently demonstrated substantial superiority over spectral measures to assess changes in HRV of OSA patients [28,29]. Therefore, both SampEn and MSE are reliable tools, able to properly show potential differences in HRV dynamics between positional and non-positional patients.

As alterations in heart rate linked to restoration of the upper airway patency have been found to be more severe during the supine position, we hypothesized that POSA patients could show an increased autonomic cardiac imbalance while sleeping. Accordingly, our aim was to assess differences in heart rate modulation between positional and non-positional patients by means of time, frequency and nonlinear indices from nocturnal pulse rate variability (PRV) recordings.

## 2. Materials and Methods

### 2.1. Participants and Sleep Studies

Patients aged ≥18 years old who visited the pneumology outpatient facilities of the Río Hortega University Hospital due to suspicion of sleep disordered breathing were asked to participate in an ambulatory study focused on the reliability of sleep apnea characterization at home. All patients showed common clinical symptoms of suffering from sleep apnea, including excessive daytime sleepiness, loud snoring, nocturnal choking and awakenings or witnessed apneas. The following exclusion criteria were considered: previous diagnosis or treatment for OSA or any other sleep disorder, including central sleep apnea and Cheyne–Stokes respiration, as well as suffering from chronic neuromuscular diseases or chronic respiratory failure. The Ethics and Clinical Research Committee of the Río Hortega University Hospital approved the protocol (CEIC 47/16; 7 April 2016). All participants signed an informed consent form, and all of the research was conducted according to the principals expressed in the Declaration of Helsinki.

Unsupervised portable polysomnography (PSG) and simultaneous nocturnal pulse oximetry (NPO) were both conducted at home. An Embletta MPR with the ST+ proxy (Embla Systems, Natus Medical Inc. Pleasanton, CA, USA) was used to perform complete PSG. An integrated triaxial accelerometer allowed us to obtain the position of the patient while sleeping. A technician programmed the device, while a trained nurse went to the patient’s home to attach all the sensors. All recordings started automatically at 23:30 p.m. and finished next day at 07:00 a.m. (450 min long). An expert in sleep research scored all PSGs to obtain the AHI and characterize OSA severity. Regarding dependence on sleeping position, the recently proposed APOC classification was used to diagnose POSA [4,9]. According to the APOC approach, a minimum amount of time (10% of total sleep time) was set in both the supine (worst) and the non-supine (best) sleeping positions in order to properly assess the influence of positional apneas. Then, OSA positive patients (total AHI ≥5 events/h) were diagnosed as having POSA if they fell into one of the following mutually exclusive categories [4,9]: (1) APOC I, or patients showing an AHI in the so-called best sleeping position (AHI_NSUP_ <5 events/h) who theoretically could be cured with positional treatment; (2) APOC II, or patients with an AHI_NSUP_ falling into a lower severity category than with the total AHI and who thus theoretically could benefit from positional treatment by decreasing its severity; and (3) APOC III, or markedly severe patients (AHI ≥40 events/h) with an AHI_NSUP_ at least 25% lower than the total AHI and who could theoretically improve their quality of life through a higher compliance to prescribed treatment.

Overnight pulse rate variability (PRV) was used as a surrogate of HRV to investigate the cardiac dynamics of POSA patients during sleep. This modality has been recently highlighted for autonomic and cardiovascular function assessment in the framework of sleep diagnosis [18]. To obtain the PRV signal, unsupervised NPO was conducted at home using a WristOx2 3150 portable pulse oximeter (Nonin Medical, Inc., Plymouth, MI, USA). The pulse oximetry device was synchronized with the polysomnograph equipment and programmed to automatically start and finish the acquisition simultaneously with the PSG study. As recommended in the framework of abbreviated testing at home [30], all NPO studies showing a total recording time (TRT) <4 h due to voluntary termination by the patient or technical issues (premature battery depletion or long loss of contact with sensor) were discarded. A pre-processing stage was implemented to detect premature beats and additional non-physiological pulse-to-pulse intervals due to a transient loss of contact with the sensor caused by the patient’s movements. Particularly, PRV samples reflecting intervals <0.33 s, >1.50 s or differing more than 0.66 s with the previous pulse-to-pulse period were removed. Finally, all 5 min segments with a >1% rate of artefacts were excluded from subsequent analyses [26,29].

### 2.2. Study Design and Sample Size

The proposed research was an ancillary study of the prospective observational ScreenOX study (NCT03295149), primarily aimed at assessing NPO as an abbreviated screening test for OSA at home. In order to ensure the statistical significance of the present secondary analysis in the context of POSA, a sample size was computed. The sample size was estimated using G*Power 3.1.9 (Düsseldorf, Germany) [31]. Differences in mean and standard deviation among POSA and non-POSA patients in previously reported HRV indices were used to measure the effect size [19]. A statistical power of 95% and a significance level of 0.05 were set, leading to a medium effect size equal to 0.45 and a minimum sample size of 216 patients.

In the ScreenOX study, a total of 320 eligible patients correctly completed both ambulatory PSG and simultaneous portable NPO. Regarding POSA, 87 studies (27.2%) were not consistent with the APOC criteria (<10% of sleep time in both non-supine and supine positions or a total AHI <5 events/h) and were removed from the study. Accordingly, 233 patients finally composed the population under study, which fit with the estimated minimum sample size. Table 1 shows the demographic and clinical characteristics of the population under study.

### 2.3. Heart Rate Modulation Assessment

Time, frequency and nonlinear analyses were conducted to thoroughly assess the PRV dynamics of POSA and non-POSA patients. Firstly, well-known pulse-to-pulse interval-based indices were computed [21]: (1) the average of the pulse-to-pulse interval (AVNN), which is a global estimate of the interbeat period (inverse of pulse rate); (2) the standard deviation of the pulse-to-pulse intervals (SDNN), which quantifies the degree of variability; and (3) the root mean square of the successive differences of pulse-to-pulse intervals (RMSSD), which accounts for vagal activity.

Despite some controversy [32,33], analysis in the frequency domain has been found to provide useful information on the modulation of heart rates by the autonomous nervous system. Accordingly, the conventional frequency bands in the framework of heart rate dynamics were characterized by the following indices [21,26]: (1) very low frequency (VLF) power (0.0033–0.04 Hz), which measures rhythms linked to the influence of the vagal and renin–angiotensin system on the pulse rate; (2) low frequency (LF) power (0.04–0.15 Hz), which captures joint modulation of the pulse rate by sympathetic and parasympathetic branches of the autonomous nervous system; (3) high frequency (HF) power (0.15–0.40 Hz), which quantifies exclusively the influence of the parasympathetic nervous system; and (4) the low frequency to high frequency ratio (LF/HF), which measures the so-called sympathovagal balance.

In order to further characterize the modulating mechanisms of the pulse rate in the frequency domain, the widely known Shannon spectral entropy (SSE) was applied to the spectrum of PRV recordings. SSE parameterizes the shape of the power spectrum of a signal so that higher SSE values account for uniform distribution along frequencies (higher irregularity in the time domain), reflecting no dominance or influence of a particular system, whereas lower SSE values are representative of a condensed spectrum in a frequency band (lower irregularity or higher periodicity in the time domain), reflecting the higher influence of a particular system [28,34]. Accordingly, the spectral entropy was computed in the whole spectrum (SSE_T_) and in the classic spectral bands of very low frequency (SSE_VLF_), low frequency (SSE_LF_), and high frequency (SSE_HF_).

Multiscale entropy (MSE) was used to assess the nonlinear dynamics of PRV in POSA and non-POSA patients. Physiological control systems, such as cardiac modulation, are characterized by complex dynamics reflecting time-dependent fluctuations. MSE is aimed at quantifying the complexity of a time series and looking for changes in entropy along different time scales [35]. To characterize the dynamical structure of a physiological recording, different coarse-grained versions of the signal were composed and further analyzed. A new version of the signal in ever deeper time scales (the so-called coarse-grained versions) was composed by averaging the samples of the original time series within non-overlapping segments of a length *τ*, increasing the window length for each new coarse-grained version. For each *τ* (i.e., for each time scale), a single-scale entropy measure was computed for the corresponding coarse-grained sequence so that the MSE curve was obtained by plotting entropy as a function of *τ* [35,36]. In this study, the well-known sample entropy (SampEn) algorithm was used to estimate the entropy [37,38]. A maximum time scale *τ* = 14 was set to ensure a proper estimation of the SampEn in the highest time scale [28,37]. According to the original work by Costa et al. [36], signals showing larger entropy values for most time scales are more complex than signals reaching lower single-scale entropies in the same region.

### 2.4. Statistical Analysis

SPSS Statistics 24 (IBM Corp., Armonk, NY, USA) and Matlab R2020a (The MathWorks Inc., Natick, MA, USA) were used to carry out statistical analyses. Overall descriptive analyses were performed in terms of the median and the 25th–75th percentile. The Kolmogorov–Smirnov normality test confirmed that the variables under study did not follow a normal distribution. Accordingly, the non-parametric Mann–Whitney test was used to assess statistical differences among non-POSA and POSA patients for quantitative continuous variables. The chi-squared test was applied for the categorical ones. In the multiclass approach (non-POSA vs. APOC I vs. APOC II vs. APOC III), the non-parametric Kruskal–Wallis test was used to assess statistical differences among groups. In addition, the Mann–Whitney test was applied to inspect differences between each particular pair of patient groups. In this regard, Fisher’s least significant difference procedure was applied to correct for multiple comparisons. All *p*-values <0.05 were considered statistically significant. Finally, linear association between the cardiac modulation indices and the polysomnographic variables was investigated using the non-parametric Spearman correlation index.

## 3. Results

A total of 129 patients were diagnosed as POSA according to APOC rules (37 APOC I, 77 APOC II and 15 APOC III), while the remaining 104 subjects comprised the non-POSA group. Table 1 shows the demographics and sleep apnea severity distribution of both groups, as well as frequent comorbidities and medications that could affect heart rate modulation.

No significant differences were found in terms of age and gender, while POSA patients showed significantly lower body mass indices (BMIs) than non-POSA subjects (27.7 vs. 29.8 kg/m^2^; *p* < 0.05). Regarding sleep apnea severity, moderate OSA was predominant among POSA patients (73.5% vs. 26.5%; *p* < 0.05), while the number of mild patients was significantly lower (37.7% vs. 62.3%; *p* < 0.05). Overall, severe patients were remarkably predominant in our sample, and there were no statistical differences between the groups under study regarding severe OSA. Finally, no significant differences were found in terms of common comorbidities and medications able to potentially influence HRV dynamics.

Table 2 summarizes the polysomnographic variables for non-POSA and POSA patients. No statistically significant differences were found between both groups concerning sleep staging. In regard to respiratory event scoring, there were no statistical differences among groups, both for total AHI and for individual apnea and hypopnea indices. On the other hand, POSA patients showed significantly lower AHIs during the rapid eye movement (REM) stage than non-POSA subjects (28.2 vs. 41.9 events/h; *p* < 0.05), as well as significantly lower AHIs in the non-supine positions (10.9 vs. 31.0 events/h; *p* < 0.001). No statistical differences were found in terms of time sleeping in the supine position and AHI values while supine. Similarly, POSA and non-POSA patients showed no significant differences in the average duration of respiratory events.

Regarding the portable unattended NPO, Table 3 shows the average values of all the variables provided by the device for the groups under study. There were no significant differences between groups concerning the oxygen desaturation indices of 3% and 4%. Regarding the hypoxemia measures, POSA patients showed significantly milder hypoxemia levels than non-POSA subjects in terms of the cumulative time with a saturation below 90% (CT90) (6.9% vs. 12.1%; *p* < 0.05) and minimum saturation (81.0% vs. 76.0%; *p* < 0.05). Similarly, POSA individuals showed slight but significantly higher average saturations than non-POSA subjects during desaturation events of both 3% (89.4% vs. 89.1%; *p* < 0.05) and 4% (88.8% vs. 88.1%; *p* < 0.05). Concerning the overall pulse rate, no statistical differences were found in terms of the average and minimum pulse rates, while POSA patients showed significantly lower maximum rates than non-POSA subjects (95.0 vs. 100.0 bpm; *p* < 0.05).

**Figure 1:**
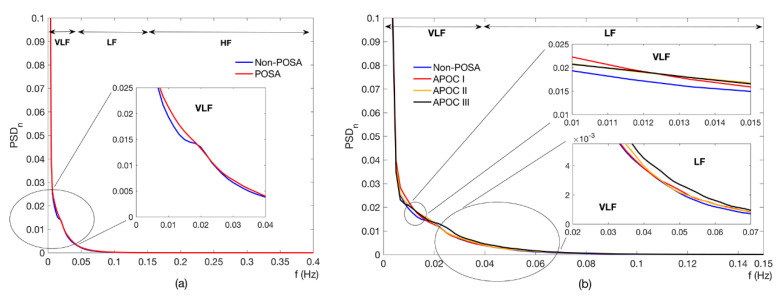
Average normalized power spectral density (PSD) curves in the classic PRV spectral bands for (**a**) non-POSA vs. POSA whole patient groups and (**b**) the four categories derived from the APOC criterion. APOC = Amsterdam POSA classification; HF = high frequency band; LF = low frequency band; POSA = positional obstructive sleep apnea; PRV = pulse rate variability; PSD_n_ = normalized power spectral density function; and VLF = very low frequency band.

**Figure 2:**
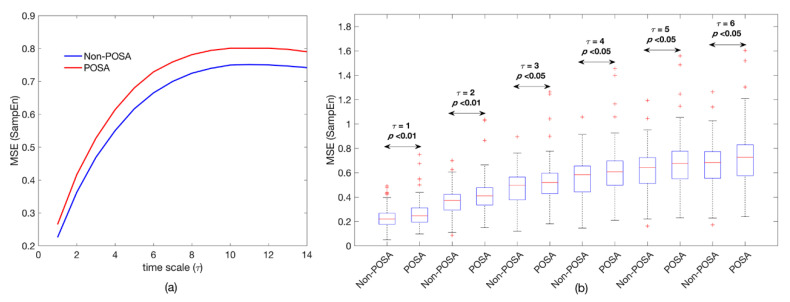
Nonlinear analysis of the nocturnal PRV recordings for non-POSA and POSA patients. (**a**) Averaged multiscale entropy (MSE) curves for the whole groups along all the time scales, and (**b**) boxplots for the statistically significant time scales. MSE = multiscale entropy; POSA = positional obstructive sleep apnea; PRV = pulse rate variability; and SampEn = sample entropy.

Table 4 summarizes the cardiac modulation indices from the long-term overnight PRV recordings of POSA and non-POSA patients. No significant differences were found between both groups regarding the conventional time and frequency domain indices. The SSE of the POSA individuals showed a slight but not significant trend toward higher irregularity in the whole spectrum (SSE_T_: 0.50 vs. 0.49; *p* = 0.056) and, particularly, in the low frequency band (SSE_LF_: 0.84 vs. 0.83; *p* = 0.062) compared with non-POSA subjects. In this way, Figure 1a illustrates the averaged power spectral content for both groups in the conventional frequency bands, where the power spectral density (PSD) curve for the POSA patients almost matched the one for the non-POSA subjects. On the contrary, nonlinear analysis by means of MSE yielded several indices able to properly parameterize the differences between the POSA and non-POSA individuals. Particularly, POSA patients showed significantly higher entropy (disorderliness) in the low time scales (*τ* ≤ 6) than the non-POSA subjects. Figure 2 shows the averaged MSE curve for each group under study. The curve for POSA patients is above the one for non-POSA subjects in all time scales, suggesting remarkably higher complexity in the overnight PRV recordings in the presence of POSA. It is important to point out that the entropy increased as the time scale also increased until a stability region was reached around *τ* = 10. This suggests that there was essential information beyond the original signal (*τ* = 1) for low time scales.

**Figure 3:**
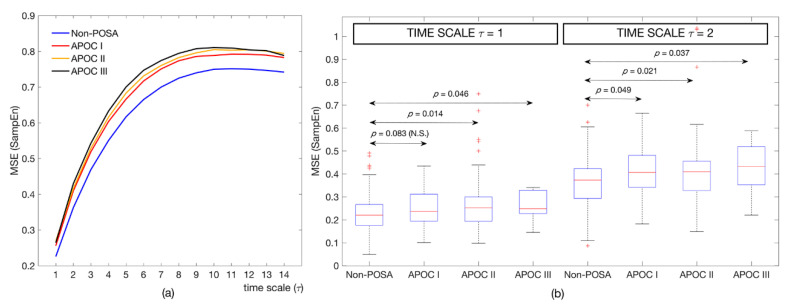
Nonlinear analysis of nocturnal PRV recordings for the four categories derived from the APOC criterion (non-POSA, APOC I, APOC II, and APOC III). (**a**) Averaged MSE curves for the whole groups along all the time scales, and (**b**) boxplots for the statistically significant time scales. APOC = Amsterdam POSA classification; MSE = multiscale entropy; POSA = positional obstructive sleep apnea; PRV = pulse rate variability; and SampEn = sample entropy.

Table 5 shows the cardiac modulation indices for the non-POSA subjects and the three POSA categories, according to the APOC criteria. No differences among groups were found using conventional measures or SSE. In the same way, it can be observed in Figure 1b that the overnight spectral content of each group was very similar, with just slight visual differences in the lower frequency bands not leading to significant *p*-values. On the contrary, MSE analysis showed significant statistical differences among the groups in the lowest time scales (*τ* ≤ 2). A pair-wise post hoc analysis yielded significant differences between the non-POSA subjects and all the POSA groups for *τ* = 2, whereas differences between the non-POSA and APOC II and III groups were found for *τ* = 1. Figure 3 shows the MSE curves for all APOC groups.

Single-entropy values along the MSE curve for low scales (*τ* ≤ 6) showed moderate but significant correlation with the total AHI (SampEn_4_: 0.235, *p* < 0.001) and AHI_SUP_ (SampEn_4_: 0.224, *p* < 0.01), higher than the conventional time domain (AVNN: −0.201, *p* < 0.01 and −0.151, *p* < 0.05, respectively) and frequency domain (LF/HF: 0.183, *p* < 0.01 and 0.197, *p* < 0.01, respectively) indices. Overall, the SSE_VLF_ yielded the highest significant correlations with the total AHI (0.365, *p* < 0.001) and AHI_SUP_ (0.350, *p* < 0.001). No scale from the MSE approach reached significant correlation with the AHI_NSUP_, whereas only the AVNN and SSE_VLF_ yielded low (−0.164, *p* < 0.05) and moderate (0.217, *p* < 0.01) correlation, respectively.

## 4. Discussion

A thorough analysis of the overnight PRV signal of positional and non-positional patients was conducted using cardiac modulation indices from different complementary approaches. Particularly, to our knowledge, this is the first study that performed a multiscale nonlinear analysis to characterize changes in nocturnal heart rate modulation due to POSA. Our analyses showed significantly higher complexity in the PRV recordings from POSA patients, compared with subjects without positional influence. Interestingly, conventional time and frequency domain indices were not able to properly characterize these differences between non-POSA and POSA individuals concerning overnight cardiac modulation. On the contrary, multiscale nonlinear analysis captured the influence of positional apneas in nighttime long-term recordings, suggesting significantly higher cardiac imbalance linked to POSA.

### 4.1. The Characteristics of POSA in Our Sample

The prevalence of POSA in our sample (55.4%) was in the lower range of that reported in the literature (56–75%) [3,4,7]. In this regard, it is important to note that all sleep studies were conducted at home, and thus POSA was diagnosed based on ambulatory PSG in the present research. As in-laboratory PSG is known to increase the time sleeping in the supine position, potentially overestimating AHI severity [2,39,40], our study may reflect a more suitable analysis of POSA and its consequences. Moreover, the prevalence of POSA in our study matches that reported in recent works using portable devices for unattended sleep apnea testing at home [2,41]. Regarding the characteristics of positional patients, they showed a slightly lower age (non-significant) and significantly lower BMI, as reported in previous studies [12,42].

Our sample showed a lower prevalence of POSA in the milder patients (37.7% vs. 62.3%) and a higher prevalence in the moderate OSA group (73.5% vs. 26.5%), while no statistical differences were found in the overall severe OSA individuals (52.7% vs. 47.3%). On the contrary, it has been commonly reported that the prevalence of POSA decreases as the severity of OSA increases, using either APOC [4,43] or additional accepted criteria for POSA [2,3,6,7,44]. However, it is important to note that mild OSA was the minority class in our population under study. In addition, our high rate of POSA among severe patients agrees with a recent study focused on the analysis of POSA characteristics in the presence of severe OSA [3] and with similar studies using the APOC criteria [4]. It is also noticeable that the APOC II patients (i.e., patients who would decrease at least one category of severity if positional apneas were removed) were predominant (59.7%) among POSA individuals in our sample, probably due to the high median overall AHI, leading to the aforementioned higher prevalence of severe OSA in the population under study.

In the context of POSA, contradictory data can be found regarding respiratory disturbance indices. In the present study, no significant differences between POSA and non-POSA individuals were found for the total AHI, apnea index, or hypopnea index (Table 1), which suggests that the increased cardiac imbalance in POSA patients is not due to a higher OSA severity degree. This agrees with previous studies [2,19], while others reported statistical differences between positional and non-positional patients regarding the total AHI [3,13]. On the other hand, non-significant differences in terms of the AHI_SUP_, as well as a significantly lower AHI_NSUP_, have been consistently reported in the literature for POSA patients compared with non-POSA subjects [3,13,19], as in the present work. Regarding the AHI during REM, Joosten et al. pointed out the potential influence of REM sleep in the generation of obstructive events [45]. Nevertheless, we found a significantly higher AHI during REM in non-POSA subjects, as in the study by Oksenberg et al. [3]. Our findings concerning NPO indices matched those previously reported in the state of the art, showing no differences in terms of oxygen desaturation indices (ODIs), while average and minimum saturations were significantly lower and CT90 was significantly higher in non-positional patients [2,13,19].

### 4.2. MSE and POSA Categories

The MSE profile was similar in both the non-POSA and POSA groups under study (Figure 1), showing gradually increasing entropy values until a region of relative stability was reached. However, there was a marked shift toward higher entropy in the POSA patients compared with the non-POSA subjects. Particularly, the MSE curve of the POSA patients was consistently above that of the non-POSA group in all time scales, thus showing higher complexity. Moreover, higher statistical differences arose in the lower time scales (*τ* ≤ 6), where the influence of apneic events was higher as the coarse-graining procedure progressively removed the respiratory-related modulation of the heart rate [36]. Regarding the three POSA groups classified according to APOC criteria (Figure 2), a consistent behavior can be observed along the scales, with remarkably lower entropy values for the non-POSA subjects and a trend toward a higher entropy as the APOC group increases in POSA patients. No statistical differences were found among APOC categories I, II, and III. Nevertheless, our analyses showed significant differences in overnight cardiac modulation between the non-POSA patients and the three POSA groups in the smallest scales of the MSE profile (*τ* ≤ 2). MSE analysis revealed that the overnight cardiac dynamics of patients in APOC categories II and III fit with those of the APOC I group, demonstrating the convenience of categorizing those patients beyond the strictly positional ones (AHI_NSUP_ < 5 events/h) as POSA because they can really benefit from positional therapy. The usefulness of MSE over traditional single-scale entropy measures applied only to the original signal maximizes in the time scale *τ* = 2, where the largest differences between groups are reached.

### 4.3. PRV Indices and Cardiac Dysfunction

It is admitted that airway obstructions are longer in the supine position, leading to deeper desaturations, longer arousals, and more severe brady-tachycardia changes compared with the lateral position [3,14,45,46]. In addition to the marked alterations in the heart rate, hypoxemia linked to the greater desaturations is related to a higher risk of cardiovascular disease and mortality [47,48]. However, POSA is commonly linked to fewer symptoms and milder disease states due to the lower overall AHI and BMI [6,9,12,13], probably underestimating the impact of positional apneas on cardiovascular regulation. In this regard, few studies investigated the potential influence of position-dependent apneas on cardiac diseases, and contradictory information exists. Favorable cardiovascular outcomes and less cardiovascular comorbidities have been suggested in positional patients [45,49,50]. Similarly, in a recent study by Byun et al. [19], POSA patients showed significantly higher parasympathetic activity (higher SDNN, RMSSD, and HF) than non-positional subjects, which had been related to reduced risk for cardiovascular disease and mortality [51,52]. In contrast, Kulkas et al. [53] reported significantly higher cardiovascular morbidity and mortality in POSA patients compared with non-positional subjects, particularly in the presence of severe OSA. Our analyses showed significantly higher randomness in the nocturnal cardiac modulation of POSA patients compared with non-POSA subjects. Higher irregularity (single-scale entropy) and complexity (multiscale entropy) are both frequently related to the greater adaptability of the autonomous nervous system and thus representative of healthier states [25,54]. However, it is important to note that entropy values are highly dependent on disease mechanisms, the conditions of recording (wakefulness vs. sleep, resting vs. exercise), and the characteristics of the time series (short-vs. long-term). As increased disorderliness of the HRV has been suggested as an independent risk factor for mortality [55], our findings are in line with the study by Kulkas et al., suggesting higher cardiac dysfunction in POSA patients. Similarly, higher entropy measures of HRV time series have been linked to increased diseased states, such as sick sinus syndrome (patient vs. healthy) [54], sleep apnea (OSA positive vs. OSA negative) [28], cardiac abnormalities (atrial fibrillation vs. healthy) [36], and overlap syndrome (COPD + OSA vs. COPD) [29]. In the same regard, Kabbach et al. recently found that COPD patients showed significantly higher variability during acute exacerbation than stable COPD patients [56]. Furthermore, in the same study, significantly higher parasympathetic activity (higher SDNN, RMSSD, and power in HF) was observed in HRV recordings from exacerbated patients, pointing out that in pathological conditions, cardiac autonomic imbalance is not exclusively associated with hyperactive sympathetic and simultaneously hypoactive parasympathetic systems, but also with increased vagal activity [51,56]. Accordingly, the higher parasympathetic activity observed by Byun et al. [19] in the HRV for POSA patients was not necessarily connected to a lower cardiovascular risk.

In the present study, the effect of POSA on cardiac regulation arose when nonlinear analysis was applied, while conventional indices were not able to detect overnight dysregulation. In this regard, it is important to point out that in the study by Byun et al., daytime short-term (5 min) HRV segments were analyzed [19], whereas overnight PRV recordings from long-term sleep studies were assessed in our research. Consequently, the influence of brady-tachycardia events during apneic episodes was actually present in our recordings. In this context, recurrent respiratory events superpose a quasi-periodic biological noise that alters autonomic cardiovascular dynamics [57]. Particularly, severe OSA has been found to introduce rhythmical fluctuations that hide the common modulation of the autonomous nervous system, notably affecting cardiac functioning [58]. Additionally, conventional indices, both in the time and in the frequency domain, are usually computed in short (≤5 min) segments to avoid non-stationary issues [21], while nonlinear MSE analyzes the entire signal, being able to capture the cumulative influence of positional apneas during the whole night beyond local or transient segments.

### 4.4. Limitations and Future Research

Some limitations should be taken into account. Firstly, there is not a standard criterion to define POSA. Several rules exist, and the literature in the context of POSA shows that there are contradictory findings concerning the characteristics and consequences of POSA potentially linked to the different criteria. Therefore, further research is needed to assess additional definitions of POSA in order to ensure the generalizability of our results. Regarding the use of PRV as a surrogate of HRV, recent studies highlighted the usefulness of PRV recordings in the assessment of autonomic cardiac function in different physiological conditions [59,60], while significant differences between both approaches were also reported [61,62] due to additional sources of variability not present in HRV [62]. Accordingly, further research is needed on the reliability of PRV analyses in the context of POSA. In addition, physiopathological mechanisms leading to the higher imbalance in overnight PRV modulation of POSA patients are not clear. The conventional CT90 and minimum saturation variables suggest higher hypoxemia in non-POSA individuals. Hypoxemia has been found to affect heart rate modulation. However, it is unknown whether nocturnal cardiac imbalance is more frequent or severe as the hypoxemia degree increases in sleep apnea patients, as well as the influence of the different OSA phenotypes on such an association. Therefore, novel measures of intermittent hypoxemia, such as the hypoxic burden, could be useful to further explain the effect of position-dependent apneic events on overnight PRV modulation. In the same regard, there is a trend toward a higher number of positional apneas in POSA patients, but a thorough analysis of individual positional apneas (event-based approach) is needed to definitely link POSA to increased cardiac dysfunction. Similarly, exhaustive research is necessary to assess whether the observed overnight imbalance becomes a continuous cardiac dysfunction in the long term. Finally, severe OSA was predominant in our sample, which could limit the generalizability of our results. Accordingly, particular analyses of overall mild and moderate OSA patients with and without positional dependence would be needed to ensure the general validity of our findings.

## 5. Conclusions

In summary, the overnight PRV recordings from POSA patients showed significantly higher complexity than those from individuals without positional dependence. This higher disorderliness points to an augmented nocturnal cardiac imbalance due to the cumulative effect of positional apneas during the whole night. Accordingly, our results suggest that POSA should not be categorized as a milder diseased state compared to non-positional sleep apnea. MSE has been found to be useful to characterize changes in nocturnal PRV modulation linked to predominant positional apneas, while conventional time and frequency domain cardiac indices were unable to detect differences between non-POSA and POSA patients.

## Figures and Tables

**Table 1:** Socio-demographic and anthropometric characteristics and sleep apnea prevalence in the population under study.

	All	Non-POSA	POSA	*p*-Value
Nº of subjects (%)	233	104 (44.6%)	129 (55.4%)	-
Nº of males (%)	167 (71.7%)	74 (71.2%)	93 (72.1%)	0.874
Age (years)	57.0 [46.0, 65.0]	57.5 [49.0, 67.0]	56.0 [44.8, 63.0]	0.058
BMI (kg/m2)	28.9 [26.1, 32.4]	29.8 [26.6, 34.7]	27.7 [26.0, 31.3]	<0.05
Sleep Apnea Prevalence				
Nº of patients 5 ≤ AHI < 15 events/h	53 (22.7%)	33 (62.3%)	20 (37.7%)	<0.05
Nº of patients 15 ≤ AHI < 30 events/h	68 (29.2%)	18 (26.5%)	50 (73.5%)	<0.05
Nº of patients AHI ≥ 30 events/h	112 (48.1%)	53 (47.3%)	59 (52.7%)	0.427
Comorbidities				
Diabetes, *n* (%)	19 (8.2%)	8 (7.7%)	11 (8.5%)	0.817
Hypertension, *n* (%)	71 (30.5%)	36 (34.6%)	35 (27.1%)	0.217
Atrial fibrillation, *n* (%)	14 (6.0%)	5 (4.8%)	9 (7.0%)	0.489
Ischemic cardiomyopathy, *n* (%)	16 (6.9%)	6 (5.8%)	10 (7.8%)	0.552
Dyslipidemia, *n* (%)	58 (24.9%)	27 (26.0%)	31 (24.0%)	0.735
COPD, *n* (%)	19 (8.2%)	12 (11.5%)	7 (5.4%)	0.090
Stroke, *n* (%)	5 (2.2%)	4 (3.9%)	1 (0.8%)	0.108
Heart failure, *n* (%)	23 (9.9%)	12 (11.5%)	11 (8.5%)	0.444
Myocardial infarction, *n* (%)	0 (0.0%)	0 (0.0%)	0 (0.0%)	-
Medications				
Beta-blockers, *n* (%)	36 (15.5%)	17 (16.4%)	19 (14.7%)	0.734
Calcium antagonists, *n* (%)	7 (3.0%)	3 (2.9%)	4 (3.1%)	0.924

Data are presented as a median [25th, 75th percentiles] or a number (percentage). BMI = body mass index; COPD = chronic obstructive pulmonary disease; and POSA = positional obstructive sleep apnea.

**Table 2:** Polysomnographic variables for Non-POSA and POSA patients.

	Non-POSA (*N* = 104)	POSA (*N* = 129)	*p*-Value
Sleep Staging			
TRT (min)	450 [449, 450]	450 [432, 450]	0.373
TST (min)	389 [354, 414]	396 [350, 419]	0.465
Sleep lat. (min)	9.7 [0.0, 22.8]	4.5 [0.0, 20.1]	0.207
N1 (%)	12.7 [7.9, 19.7]	10.9 [6.4, 16.2]	0.054
N2 (%)	36.3 [30.0, 44.4]	34.8 [30.3, 42.7]	0.479
N3 (%)	27.4 [19.4, 33.5]	28.4 [21.5, 34.0]	0.202
REM (%)	22.4 [17.4, 26.7]	23.7 [19.1, 27.4]	0.111
REM lat. (min)	71.3 [46.3, 108.3]	72.5 [45.3, 95.6]	0.427
Respiratory Event Scoring			
AI (events/h)	5.8 [1.7, 22.7]	4.6 [1.4, 12.5]	0.096
HI (events/h)	19.1 [10.3, 32.2]	19.9 [12.9, 28.7]	0.845
AHI (events/h)	32.5 [13.1, 63.0]	27.7 [17.6, 41.3]	0.160
AHI_REM_ (events/h)	41.9 [23.4, 65.2]	28.2 [15.8, 54.5]	<0.05
AHI_NREM_ (events/h)	28.9 [11.3, 60.9]	25.0 [14.1, 38.8]	0.153
AHI_SUP_ (events/h)	41.3 [16.3, 69.4]	51.0 [31.8, 67.9]	0.067
AHI_NSUP_ (events/h)	31.0 [10.0, 55.2]	10.9 [4.3, 24.1]	<0.05
TS_SUP_ (%)	40.9 [27.7, 58.8]	44.4 [31.6, 58.8]	0.352
T_AVG_ event (s)	23.5 [21.0, 26.6]	22.9 [20.9, 26.1]	0.793
ArI_TOT_ (events/h)	21.9 [13.1, 37.4]	19.1 [12.1, 25.5]	0.056
ArI_RESP_ (events/h)	12.7 [5.3, 29.9]	11.6 [7.2, 17.1]	0.247

Data are presented as a median [25th, 75th percentiles]. AHI = apnea-hypopnea index; AHI_NREM_ = apnea-hypopnea index during non-REM sleep; AHI_NSUP_ = apnea-hypopnea index while sleeping in a non-supine position; AHI_REM_ = apnea-hypopnea index during REM sleep; AHI = apnea-hypopnea index while sleeping in a supine position; AI = apnea index; ArI_RESP_ = respiratory arousal index; ArI_TOT_ = total arousal index; HI = hypopnea index; N1–N3 = percentage of time in the N1, N2 and N3 sleep stages; OSA = obstructive sleep apnea; POSA = positional obstructive sleep apnea; REM = percentage of time in the rapid eye movement sleep stage; Sleep lat = sleep latency; T_AVG_ event = average duration of respiratory events; TS_SUP_ = total sleep time in a supine position; TRT = total recording time; and TST = total sleep time.

**Table 3:** SpO_2_ and pulse rate variables from portable pulse oximetry for non-POSA and POSA patients.

	Non-POSA (*N* = 104)	POSA (*N* = 129)	*p*-Value
TRT (min)	450 [450, 450]	450 [450, 450]	0.337
ODI3 (events/h)	24.1 [13.1, 49.9]	23.3 [14.3, 36.4]	0.110
ODI4 (events/h)	13.3 [6.1, 37.7]	14.2 [6.7, 22.3]	0.093
CT90 (%)	12.1 [2.9, 40.0]	6.9 [1.0, 17.2]	<0.05
SpO2_MIN_ (%)	76.0 [69.5, 81.5]	81.0 [76.0, 85.0]	<0.05
SpO2_AVG(noEv3%)_ (%)	92.1 [90.5, 93.4]	92.6 [91.2, 93.7]	0.079
SpO2_AVG(noEv4%)_ (%)	92.1 [90.3, 93.2]	92.5 [91.2, 93.5]	0.068
SpO2_AVG(inEv3%)_ (%)	89.1 [86.5, 90.5]	89.4 [88.1, 90.8]	<0.05
SpO2_AVG(inEv4%)_ (%)	88.1 [85.5, 89.6]	88.8 [87.5, 90.1]	<0.05
PR_AVG_ (bpm)	63.9 [58.0, 69.6]	62.8 [56.2, 68.2]	0.108
PR_MIN_ (bpm)	46.0 [39.0, 51.0]	47.0 [40.8, 52.0]	0.429
PR_MAX_ (bpm)	100.0 [90.0, 111.5]	95.0 [86.0, 108.3]	<0.05

Data are presented as a median [25th, 75th percentiles]; bpm = beats per minute; CT90 = cumulative time with saturation below 90%; ODI3 = oxygen desaturation index of 3%; ODI4 = oxygen desaturation index of 4%; PR_AVG_ = average pulse rate; PR_MAX_ = maximum pulse rate; PR_MIN_ = minimum pulse rate; SpO2_AVG(inEv3%)_ = average blood oxygen saturation in desaturations >3%; SpO2_AVG(inEv4%)_ = average blood oxygen saturation in desaturations >4%; SpO2_AVG(noEv3%)_ = average blood oxygen saturation removing desaturations >3%; SpO2_AVG(noEv4%)_ = average blood oxygen saturation removing desaturations >4%; SpO2_MIN_ = minimum blood oxygen saturation; and TRT = total recording time.

**Table 4:** Pulse rate variability (PRV)-derived cardiac indices for non-POSA and POSA patients.

	Non-POSA (*N* = 104)	POSA (*N* = 129)	*p*-Value
Time Domain Indices			
AVNN (ms)	0.95 [0.88, 1.05]	0.97 [0.89, 1.08]	0.148
SDNN (ms)	0.04 [0.03, 0.06]	0.04 [0.03, 0.05]	0.581
RMSSD (×10^−4^) (ms)	5.99 [4.89, 7.48]	6.19 [4.78, 7.50]	0.679
Frequency Domain: Relative Power			
P_T_ (1/Hz)	3.75 [2.10, 5.30]	3.40 [2.08, 4.99]	0.396
VLFn (nu)	0.39 [0.31, 0.50]	0.40 [0.31, 0.51]	0.544
LFn (nu)	0.94 [0.91, 0.96]	0.93 [0.91, 0.95]	0.761
HFn (nu)	0.06 [0.05, 0.09]	0.07 [0.05, 0.09]	0.760
LF/HF (nu)	14.55 [9.60, 21.40]	13.06 [9.86, 19.48]	0.761
Frequency Domain: Spectral Entropy			
SSE_T_ (nu)	0.49 [0.47, 0.51]	0.50 [0.48, 0.52]	0.056
SSE_VLF_ (nu)	0.93 [0.90, 0.95]	0.93 [0.91, 0.96]	0.147
SSE_LF_ (nu)	0.83 [0.80, 0.85]	0.84 [0.81, 0.86]	0.062
SSE_HF_ (×10^−1^) (nu)	9.70 [9.65, 9.77]	9.70 [9.65, 9.76]	0.998
Nonlinear Analysis: Multiscale Entropy			
SampEn_1_ (nu)	0.22 [0.18, 0.27]	0.25 [0.20, 0.31]	<0.05
SampEn_2_ (nu)	0.37 [0.29, 0.42]	0.41 [0.34, 0.48]	<0.05
SampEn_3_ (nu)	0.50 [0.38, 0.57]	0.52 [0.43, 0.60]	<0.05
SampEn_4_ (nu)	0.58 [0.44, 0.66]	0.61 [0.50, 0.70]	<0.05
SampEn_5_ (nu)	0.64 [0.51, 0.72]	0.68 [0.55, 0.78]	<0.05
SampEn_6_ (nu)	0.68 [0.55, 0.77]	0.73 [0.58, 0.83]	<0.05
SampEn_7–14_ (nu)	0.73 [0.56, 0.83]	0.77 [0.61, 0.89]	0.087

Data are presented as a median [25th, 75th percentiles]. AVNN = average of the pulse-to-pulse interval; HFn = normalized spectral power in the high frequency band; LF/HF = low frequency to high frequency ratio or sympathovagal balance; LFn = normalized spectral power in the low frequency band; nu = normalized units; OSA = obstructive sleep apnea; POSA = positional obstructive sleep apnea; P_T_ = total signal power; RMSSD = root mean square of successive differences of the pulse-to-pulse intervals; SampEn_j_ = sample entropy in the scale *τ* = *j*; SDNN = standard deviation of the pulse-to-pulse interval; SSE_T_ = spectral entropy in the whole spectra; SSE_VLF_ = spectral entropy in the very low frequency band; SSE_LF_ = spectral entropy in the low frequency band; SSE_HF_ = spectral entropy in the high frequency band; VLFn = normalized spectral power in the very low frequency band.

**Table 5:** PRV-derived cardiac indices among the POSA categories, according to the APOC criteria.

	Non-POSA (*N* = 104)	APOC I (*N* = 37)	APOC II (*N* = 77)	APOC III (*N* = 15)	*p*-Value
Time Domain Indices					
AVNN (ms)	0.95 [0.88, 1.05]	0.96 [0.91, 1.05]	0.98 [0.89, 1.09]	0.96 [0.80, 1.13]	0.455
SDNN (ms)	0.04 [0.03, 0.06]	0.04 [0.03, 0.05]	0.04 [0.03, 0.05]	0.04 [0.03, 0.056	0.751
RMSSD (×10^−4^) (ms)	5.99 [4.89, 7.48]	5.83 [4.56, 7.16]	6.25 [4.90, 7.63]	6.78 [4.72, 8.31]	0.758
Frequency Domain: Relative Power					
P_T_ (1/Hz)	3.75 [2.10, 5.30]	3.09 [2.10, 4.18]	3.76 [2.11, 5.12]	3.60 [1.84, 6.15]	0.626
VLFn (nu)	0.39 [0.31, 0.50]	0.42 [0.33, 0.47]	0.40 [0.30, 0.52]	0.44 [0.30, 0.51]	0.924
LFn (nu)	0.94 [0.91, 0.96]	0.94 [0.92, 0.95]	0.92 [0.90, 0.95]	0.94 [0.91, 0.95]	0.610
HFn (nu)	0.06 [0.05, 0.09]	0.06 [0.05, 0.08]	0.08 [0.05, 0.10]	0.06 [0.05, 0.09]	0.609
LF/HF (nu)	14.55 [9.60, 21.40]	15.96 [11.10, 20.55]	12.20 [8.97, 19.48]	15.07 [9.99, 19.18]	0.610
Frequency Domain: Spectral Entropy					
SSE_T_ (nu)	0.49 [0.47, 0.51]	0.50 [0.49, 0.51]	0.50 [0.48, 0.52]	0.51 [0.49, 0.53]	0.195
SSE_VLF_ (nu)	0.93 [0.90, 0.95]	0.93 [0.91, 0.95]	0.93 [0.91, 0.96]	0.94 [0.93, 0.96]	0.302
SSE_LF_ (nu)	0.83 [0.80, 0.85]	0.83 [0.82, 0.85]	0.84 [0.81, 0.86]	0.84 [0.80, 0.87]	0.298
SSE_HF_ (×10^−1^) (nu)	9.70 [9.65, 9.77]	9.72 [9.66, 9.75]	9.70 [9.65, 9.76]	9.65 [9.61, 9.78]	0.936
Nonlinear Analysis: Multiscale Entropy					
SampEn_1_ (nu)	0.22 [0.18, 0.27] ^⁋,†^	0.24 [0.19, 0.31]	0.25 [0.19, 0.30] ^⁋^	0.25 [0.23, 0.33] ^†^	<0.05
SampEn_2_ (nu)	0.37 [0.29, 0.42] *^,⁋,†^	0.41 [0.34, 0.48] *	0.41 [0.33, 0.46] ^⁋^	0.43 [0.35, 0.52] ^†^	<0.05
SampEn_3_ (nu)	0.50 [0.38, 0.57]	0.51 [0.44, 0.60]	0.52 [0.43, 0.59]	0.56 [0.44, 0.67]	0.082
SampEn_4–14_ (nu)	0.72 [0.55, 0.82]	0.77 [0.61, 0.88]	0.75 [0.61, 0.88]	0.77 [0.62, 0.91]	0.360

Data are presented as a median [25th, 75th percentiles]; nu = normalized units; AVNN = average of the pulse-to-pulse interval; HFn = normalized spectral power in the high frequency band; LF/HF = low frequency to high frequency ratio or sympathovagal balance; LFn = normalized spectral power in the low frequency band; OSA = obstructive sleep apnea; POSA = positional obstructive sleep apnea; P_T_ = total signal power; RMSSD = root mean square of the successive differences of the pulse-to-pulse intervals; SampEn*_j_* = sample entropy in the scale *τ* = *j*; SDNN = standard deviation of the pulse-to-pulse interval; SSE_T_ = spectral entropy in the whole spectra; SSE_VLF_ = spectral entropy in the very low frequency band; SSE_LF_ = spectral entropy in the low frequency band; SSE_HF_ = spectral entropy in the high frequency band; VLFn = normalized spectral power in the very low frequency band. * Significant differences in non-POSA vs. APOC I subjects; ^⁋^ Significant differences in non-POSA vs. APOC II subjects; ^†^ Significant differences in non-POSA vs. APOC III subjects; ^‡^ Significant differences in APOC I vs. APOC II subjects; ^╫^ Significant differences in APOC I vs. APOC III subjects; ^⸸^ Significant differences in APOC II vs. APOC III subjects.
